# Saliency mapping in the optic tectum and its relationship to habituation

**DOI:** 10.3389/fnint.2014.00001

**Published:** 2014-01-16

**Authors:** Arkadeb Dutta, Yoram Gutfreund

**Affiliations:** Rappaport Family Institute for Research in the Medical Sciences, Ruth and Bruce Rappaport Faculty of Medicine, Technion – Israel Institute of TechnologyHaifa, Israel

**Keywords:** habituation, saliency map, optic tectum, superior colliculus, spatial attention, barn owl, orienting response

## Abstract

Habituation of the orienting response has long served as a model system for studying fundamental psychological phenomena such as learning, attention, decisions, and surprise. In this article, we review an emerging hypothesis that the evolutionary role of the superior colliculus (SC) in mammals or its homolog in birds, the optic tectum (OT), is to select the most salient target and send this information to the appropriate brain regions to control the body and brain orienting responses. Recent studies have begun to reveal mechanisms of how saliency is computed in the OT/SC, demonstrating a striking similarity between mammals and birds. The saliency of a target can be determined by how different it is from the surrounding objects, by how different it is from its history (that is habituation) and by how relevant it is for the task at hand. Here, we will first review evidence, mostly from primates and barn owls, that all three types of saliency computations are linked in the OT/SC. We will then focus more on neural adaptation in the OT and its possible link to temporal saliency and habituation.

## INTRODUCTION: HABITUATION AND SALIENCY MAPPING

An animal can respond to only one stimulus source at a time, even though its sensory systems are bombarded by information arriving from multiple sources. This unequal relationship between response and stimulus numbers explains the evolvement of brain mechanisms to identify and select the most appropriate stimulus for behavioral manifestation. The process, commonly known as selective attention, saliency mapping, or sensory gating (Itti and Koch, 2001; Krauzlis et al., 2013), is a fundamental characteristic of human and animal behavior. Failing to choose the appropriate stimulus severely disrupts normal behavior, as can be seen in the various attention deficits disorders (Gitelman, 2003; Booth et al., 2005).

How can an animal decide if a stimulus is behaviorally relevant? What are the sensory cues and to what extent are they general across species? A dog among cats, a brown object among orange objects, or a pure tone succeeding a long period of broadband noise are all conspicuous stimuli. Such stimuli are perceived as salient and consequently behaviorally privileged as they give rise to attentional capture (Posner, 1980; Tiitinen et al., 1994), enhanced autonomic responses (Weisbard and Graham, 1971; Bala and Takahashi, 2000; Zimmer, 2006), and enhanced neural responses (Naatanen, 1995; Gottlieb et al., 1998; Ulanovsky et al., 2003; Reches and Gutfreund, 2008). Thus, a general rule is that a stimulus that is out-of-ordinary (unexpected) is salient and has a higher probability to induce responses. In nature, an out-of-ordinary event may come as a warning to prey to evade danger or an opportunity for food in the case of predators. Therefore, rapid detection of such unexpected events is fundamental for survival. A stimulus can be out-of-ordinary in space, as in the first two examples above, or out-of-ordinary in time, as in the third example above. The process of detecting stimuli that are out-of-ordinary in space is called stimulus competition, spatial saliency mapping, or camouflage breaking (Itti and Koch, 2000; Knudsen, 2007). The process of detecting stimuli that are out-of-ordinary in time is called temporal saliency mapping, deviance detection, or change detection (Nelken and Ulanovsky, 2007; Gutfreund, 2012).

Imagine a dog resting in the center of a noisy living room, seemingly ignoring the loud noise of kids, and music playing but immediately raising its head to the faint sound of the door knob turning. Note that the event of the door knob turning is not louder than the background noise. This example demonstrates a major aspect of saliency mapping; that the saliency of a stimulus is not determined by its physical strength but by its relationship with the environment, or, in other words, by its context. The door knob turning is a salient event mainly because it breaks the regularity of the background and is therefore unexpected.

**FIGURE 1:**
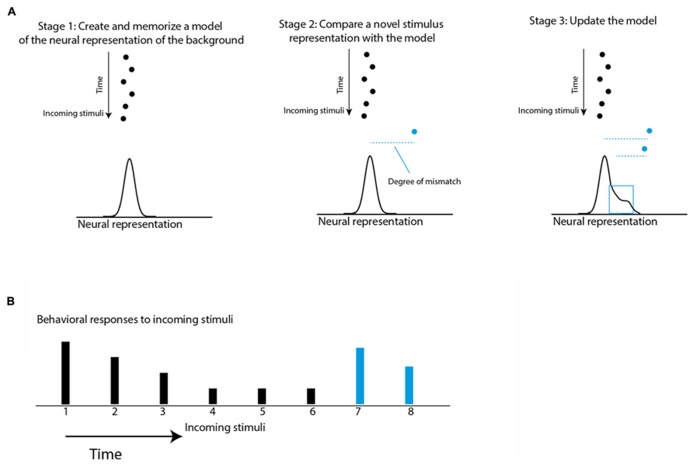
**Schematic illustration of a conceptual model of habituation and saliency mapping.** **(A)** The black dots represent a stream of stimuli coming one after the other in time. The horizontal positions represent points on the internal neural representation axis. The statistics of the incoming stimuli are learned and memorized in Stage 1 to create an expectation model that is the expected probability of the internal representation of the next stimulus. The expectation model is illustrated by a normal curve. The blue dot is a new incoming stimulus. In Stage 2, the expectation violation of the new stimulus is measured based on the degree of mismatch between the internal representation of the new stimulus and the memorized model (dashed blue line). Then, in Stage 3 the representation of the new stimulus is incorporated into the model to affect the degree of mismatch of further incoming stimuli. **(B)** The height of the bars represents the predicted behavioral responses to the incoming stimuli (dots in **A**) based on the assumption that each response is proportional to the degree of mismatch.

In the case of temporal saliency, as in the door knob example, an initial phase of learning and memorizing the regularity of the background must take place so that an incoming stimulus can be categorized as either background or deviant. Responses to stimuli matching the background are suppressed while responses to deviants from the background are not. **Figure 1** illustrates a conceptual model for temporal saliency mapping. Interestingly, a similar concept was used to model habituation of the orienting reflex (Sokolov, 1963; Siddle, 1991). Thus, there seems to be a considerable overlap between habituation and saliency mapping. Habituation is considered the most basic form of learning, existing in all animals (Sokolov, 1963; Thompson and Spencer, 1966; Thompson et al., 1972; Barry, 2009; Thompson, 2009). Although habituation has been described and studied in detail for decades, most of the previous studies on habituation were separated from studies on saliency mapping and attention (Dukewich, 2009). In this review, we aim at emphasizing the close relationship prevailing between habituation and saliency mapping and the likelihood that there is a considerable overlap between the neural mechanisms of the two processes.

In addition to habituation, associative learning may also be involved in saliency mapping (Anderson, 2013). It is obvious that the successful selection of stimuli cannot be based on external factors alone. Selection of a stimulus must be guided by a combination of external factors, such as stimulus intensity, stimulus history, spatial context, cross-modal interactions, etc., and internal factors, such as cognitive biases, behavioral tasks, reward history, motivations, etc. (Fecteau and Munoz, 2006). For example, when searching for a friend in crowd, a memorized knowledge such as shirt color or hair type biases the perceived saliency of stimuli. Such information about the relevance of the stimulus for the task at hand is called “top-down” information as opposed to information about the sensory aspects of the stimulus, which is called “bottom-up” information. Somewhere in the brain, top-down information must be integrated with bottom-up information to determine the saliency of the event (Fecteau and Munoz, 2006). Where in the brain this integration happens and what are the mechanisms involved are open questions. It is possible that top-down information can modulate the internal representation of the background created by the bottom-up stream (illustrated in **Figure 1**). This hypothesis is attractive as it implies that cognitive factors may influence stimulus selection by recruiting the same brain structures that are involved in temporal saliency and habituation. We will hypothesize here and show evidence that brain areas or networks that select stimuli for behavior are likely to show neural correlates of habituation. The brain structure that we will focus on is the optic tectum (OT), also known as the superior colliculus (SC) in mammalian species. We will first review evidence supporting a role of the OT/SC in both spatial and temporal saliency mapping and then focus more on temporal saliency mapping and its relation to habituation (for more comprehensive reviews on spatial saliency mapping see Itti and Koch, 2001; Mysore and Knudsen, 2011b).

## SALIENCY MAPPING IN THE OT/SC

**FIGURE 2:**
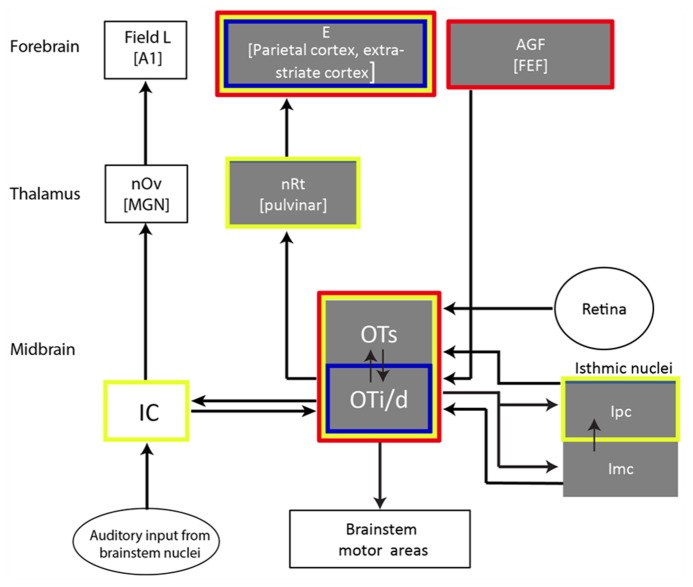
**Illustration showing a selected part of the barn owl’s auditory pathway with the OT as a hub.** Gray boxes indicate structures suggested to be related to gaze and attention control. The red outline indicates structures where SSA was reported; the blue outline indicates areas where long-term adaptation was reported (habituation-like responses); the yellow outline indicates structures where multisensory (visual/auditory) neurons were reported. Double-headed arrows indicate reciprocal connections. Abbreviations of names of structures appear within each box. The abbreviations of names of the equivalent mammalian structures are in parentheses: E, entopallium; AGF, arcopallium gaze fields; FEF, frontal eye fields; nRt, nucleus rotundus; OTs, superficial layers of the OT (layers 1–10); OTi/s, intermediate and deep layers of the OT (layers 11–15); IC, inferior colliculus; Ipc, nucleus isthmi pars parvocellularis; Imc, nucleus isthmi pars magnocellularis; nOv, nucleus ovoidalis; MGN, medial geniculate body; A1, auditory cortex 1.

All vertebrates possess a specialized brain system responsible for orienting the body toward stimuli of interest. This system, known as the gaze control system (also referred to as the oculomotor system), involves a number of midbrain and forebrain areas (see scheme of basic avian circuitry in **Figure 2**). The OT/SC is a midbrain structure serving as a critical hub in the gaze control system (Boehnke and Munoz, 2008). This structure is arguably one of the most phylogenetically conservative structures in the brain (Gaither and Stein, 1979; Shimizu and Karten, 1993; Luksch, 2003) and is considered homolog in all vertebrate species (Butler and Hodos, 2005; Maximino, 2008).

Both mammalian SC and avian OT contain a mapped representation of space. This map is multimodal with neurons responding to auditory, visual, and somatosensory signals (Knudsen, 1982; King and Palmer, 1985; Stein and Meredith, 1993; Nodal et al., 2005). The sensory map of space is superimposed over a motor map controlling gaze shifts (McHaffie and Stein, 1982; Sparks, 1986; Herrero et al., 1998; King, 2004). Hence, the OT/SC is known primarily as a gaze control center serving to translate sensory signals to eye and head movements. However, a careful examination of the anatomy with a detailed electrophysiological characterization of the neural responses suggests that the OT/SC is more than a simple motor system. In addition to its control of pre-motor areas, information from the OT/SC is also transmitted to a wide range of areas in the cortex and basal ganglia via thalamic nuclei (Takada et al., 1985; Robinson and Petersen, 1992; Bischof and Watanabe, 1997; Reches and Gutfreund, 2009; Krauzlis et al., 2013), indicating involvement in non-motor functions as well. The emerging hypothesis is that the evolutionary role of the OT/SC is to sort stimuli based on saliency, select the most salient stimulus, and send this information to the appropriate brain regions to direct orienting movements, attention and autonomic responses (reviewed in Boehnke et al., 2011; Knudsen, 2011; Krauzlis et al., 2013). In the following sections, we will review some evidence supporting this hypothesis.

Many studies suggest that neurons in the OT/SC are not simply sensitive to the intensity of the stimulus inside their receptive field (RF) but rather to the saliency of the position represented by their RF at a given instant (Horwitz and Newsome, 1999; McPeek and Keller, 2002; Pluta et al., 2011). This suggests that OT/SC plays a role in target selection (a target is a stimulus worth responding to). For example, in a study to show the role of SC in target selection for saccade execution, Horwitz and Newsome (1999) trained monkeys to choose one of two visual targets based upon the perceived direction of moving dots in a random-dot motion display around the center of fixation. One of the targets was presented inside the RF of the recorded SC neuron and the other well outside the field. Neural recordings revealed that some neurons in the SC responded to the target in their RF only if the direction of the dots was pointing to the direction of their RF location, thus, selecting the target well before the execution of the saccades. Moreover, in well trained monkeys, some neurons responded even without visual stimuli inside their RF, provided that the motion of the dots at the center is contingent with their RF (Horwitz and Newsome, 2001). One interpretation of these results is that the neurons represent the saliency of the stimulus inside their RFs. The learned association between the motion of the dots at the center and the rewarded target enhances the saliency of the position of the RFs pointed out by the motion and, on the other hand, reduces the saliency of the positions not pointed out by the central display.

Further compelling evidence for the causal role of the SC in stimulus selection was provided by inactivation experiments. When a restricted part of the SC is inactivated, monkeys tend to miss the behaviorally relevant targets if they are positioned in the region represented by the inactivated area. Instead, the monkeys choose distracters in areas whose representation is unaffected by the inactivation (McPeek and Keller, 2004). Lovejoy and Krauzlis (2009) expanded this finding to show that focal inactivation of the SC also disrupts the monkey’s ability to select stimuli covertly in the inactivated regions (Krauzlis et al., 2013). Interestingly, in both studies, when the stimulus in the inactivated region was presented alone, the monkeys were able to respond and covertly attend to the stimulus (McPeek and Keller, 2004; Krauzlis et al., 2013), thus, focal inactivation of the SC does not create a focal sensory neglect. Only in situations when multiple stimuli are presented are the inactivation effects apparent. Thus, SC inactivation specifically disrupts the ability to select the behaviorally relevant stimulus among other non-relevant stimuli.

Analogous results pointing out the importance of the OT/SC in stimulus selection have been reported in other species as well (Ingle, 1975; Woods and Frost, 1977; Marin et al., 2007; Lai et al., 2011; Pluta et al., 2011). Of particular interest here is a series of recent studies in barn owls addressing mechanisms of competitive stimulus selection in the OT (Mysore et al., 2010, 2011; Mysore and Knudsen, 2011b). A stimulus presented alone commonly induces responses in the OT/SC that are larger compared to when it is presented together with other stimuli. This phenomenon, which has been attributed to lateral inhibition, may be interpreted as promoting competition between stimuli (McPeek and Keller, 2002; Knudsen, 2011). Interestingly, Mysore et al. (2010, 2011) have shown some novel properties of this lateral competitive interaction. First, the strength of the suppression does not depend on the distance between the stimuli, which shows that it is a global phenomenon covering the whole visual field (Mysore et al., 2010). Second, many of the neurons are suppressed by competing stimuli only if the strength of the stimulus inside their RF is weaker from the other stimuli but are not suppressed if the RF contains the strongest stimulus in the scene (Mysore et al., 2011). Thus, it seems that the OT tends to code the strength of a stimulus relative to its competitors, a feature that may promote a winner-take-all computation (Mysore and Knudsen, 2011a). Moreover, the stimulus representation in the OT is modulated by top-down connections from forebrain areas (Winkowski and Knudsen, 2006, 2007), thus, possibly allowing for the selection of stimuli not only via their relative strengths but also incorporating cognitive factors such as learned associations, internal states, etc. The mechanism of lateral competitive interactions in the barn owl OT were shown to be mediated by a GABAergic midbrain nucleus (Imc, nucleus isthmi pars magnocellularis) that receives topographic connections from the OT directly and indirectly through a nearby cholinergic nucleus (Mysore and Knudsen, 2013). Thus, the computation of stimulus selection is, at least partly, achieved within the tectal circuitry.

The results described above, as well as numerous other studies, support the above-mentioned hypothesis about the role of the OT/SC as a center of stimulus selection, and shows that this role is preserved across species and across sensory modalities. Two questions, however, remain to be answered. The first question is whether all these elaborate mechanisms of stimulus selection in the OT/SC evolved just for the purpose of gaze and attention. An event that is perceived by an animal as salient typically induces a wide range of behavioral responses (Sokolov, 1963; Barry, 2009; Bradley, 2009). Although a shift in gaze is the most apparent response, a series of autonomic reflexes occur along with it that prepares the body for possible action (Sokolov, 1963; Dean et al., 1989). These include galvanic responses (Bradley, 2009), changes in heart rate (Bradley, 2009), changes in brain wave activity (Naatanen, 1995), and pupillary dilation (Oleson et al., 1972; Stelmack and Siddle, 1982; Bala and Takahashi, 2000). This wide repertoire of responses, which is preserved remarkably across species, has been coined by Ivan Pavlov as the “orienting response” (Sokolov, 1963). Orienting responses can include gaze shifts (overt orienting), but do not have to (covert orienting). In addition, orienting movements can be executed by locomotory muscles of limbs instead of eyes. Therefore, if the hypothesis regarding the role of OT/SC as a center of saliency mapping holds true, then it is predicted that manipulation of activity in the OT/SC will affect orienting responses in general beside eye movements. Evidence supporting this prediction can be found in the literature: microstimulation in the OT/SC can induce pupil dilation responses (PDRs; Netser et al., 2010; Wang et al., 2012); responses in the EMG activity of neck muscles independent of eye or head movements (Corneil et al., 2007); ocular accommodation (Sawa and Ohtsuka, 1994); freezing responses (Dean et al., 1989); increased heart rate (Keay et al., 1988); arousal in cortical EEG (Redgrave and Dean, 1985); and suppression of eye blink reflex (Basso, 1996; Gnadt et al., 1997). Thus, it is evident that apart from its clear role in controlling eye and head movements, the OT/SC is also widely involved in executing a variety of orienting behaviors.

**FIGURE 3:**
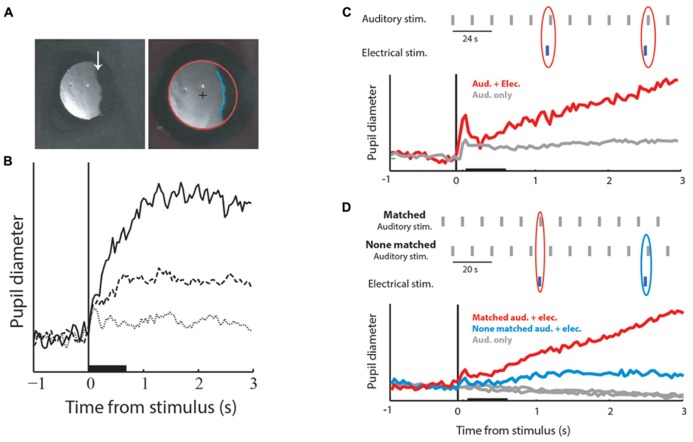
**Pupil dilation responses (PDRs) and microstimulation in the OT.** **(A)** The left image shows a zoomed image of a barn owl’s right eye. The image is a video frame taken from an infra-red sensitive video sequence showing the infra-red light reflected from the retina. The arrow points to the edge of the pecten, a retinal landmark absorbing light. The image on the left shows a similar video frame after processing and fitting the pupil edge with an ellipse. The red circle designates the fitted pupil edge. The cross designates the center of the circle and the blue line shows the horizontal edge of the pecten. The diameter of the circle and the average horizontal position of the pecten edge were used to measure pupil dilations and eye movements. **(B)** The graph shows PDRs to three consecutive auditory stimuli given every 12 s. The solid line designates the response to the first stimulus, the dashed line the response to the second stimulus and the dotted line the response to the third stimulus. The base line of all response profiles was reduced to zero level. The horizontal bar indicates the duration of the acoustic stimulus and the vertical line the onset of stimulation. **(C)** Results of coupling acoustic and tectal electrical stimulation. The inset shows the time course of the stimulation protocol. Auditory stimuli were repeated every 12 s (gray bars). Occasionally, with a probability of 20%, a brief low-level microstimulation was injected shortly before the auditory stimulus (blue bars). The gray curve shows the population average PDR to the repeated auditory stimulus. The red curve shows the population average PDR to the auditory stimuli that followed a microstimulation. Note the release from habituation. **(D)** The inset shows the time course of the experiment. The gray vertical bars indicate auditory stimuli and the blue vertical bars electrical stimulations. Auditory stimuli were repeated every 10 s alternating between two positions: one matching the electrical stimulation site in the tectal map and the other not matching the electrical stimulation site. The gray curves show the population PDRs to the two auditory stimuli. The blue curve shows the population PDR to the non-matched auditory stimuli that were coupled with the microstimulation, and the red curve the population PDR to the matched auditory stimuli coupled with the microstimulation. Microstimulation at the site corresponding with the acoustic stimulus induces a stronger release from habituation. Modified from Netser et al. (2010).

The second question is whether the OT/SC is also involved in temporal saliency detection. As discussed in the introduction, a major part of saliency mapping is history-dependent, that is, a stimulus that is different from its past, is likely to be perceived as salient. However, all of the papers cited above as evidence for the involvement of the OT/SC in saliency mapping, emphasize situations of spatial saliency where the saliency of the target is determined by the difference from the surround. If the OT/SC is indeed a center of saliency mapping in the brain, then it is likely to be involved in the habituation process. Hence, we predict that manipulating tectal activity will disrupt habituation of orienting responses. An indication that indeed this is the case has been provided by Netser et al. (2010). The pupils of barn owls, no different from other species, dilate slightly in response to sudden sounds (**Figure 3A**; Bala and Takahashi, 2000; Spitzer et al., 2003). Netser et al. (2010) measured the pupil diameter of barn owls exposed to a long sequence of identical auditory stimuli. As a result of the long period of repetition, the PDRs became habituated (**Figure 3B**). However, it was shown that if a brief low-level electrical microstimulation is applied to the OT at the site in the map corresponding to the location of the stimulus, the habituated behavioral responses are re-induced (**Figure 3C**). This could not be attributed to general desensitization by the microstimulation as it was significantly less induced by stimulations at other locations in the map (**Figure 3D**). This indicates that the release from habituation was due to manipulations at the local tectal circuitry and therefore supports its involvement in habituation.

Given its suggested role in habituation of the orienting response, we expect to find neural correlates of habituation in the OT/SC. In other words, we expect that neural representation will be strongly modulated by the history of events in a way that suppresses the representation of background stimuli and relatively enhances the representation of odd stimuli. In the following sections, we will discuss the requirements for neural correlates of habituation and then review the literature suggesting such correlates in the OT of the barn owl.

## NEURAL CORRELATES OF HABITUATION

**FIGURE 4:**
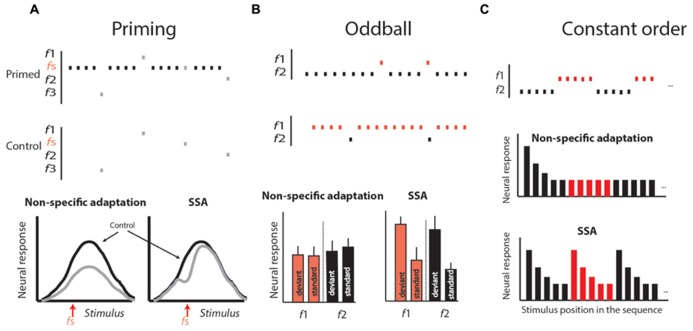
**Stimulus protocols used to differentiate stimulus-specific adaptation (SSA) from non-specific adaptation.** **(A)** The priming protocol. In the priming protocol a tuning curve is generated by presenting sequences of stimuli varying randomly over a range of stimuli (f1–fn). This is repeated in two conditions: in one, the stimuli for generating the tuning curve are spaced with a fixed time interval of no stimulation (the control); in the other, in between stimuli, a standard stimulus (fs) is presented several times. In non-specific adaptation (left graph), the tuning curve in the primed condition (gray curve) is expected to be attenuated evenly relative to the control. In SSA, the adaptation is expected to occur stronger near the fs stimulus, resulting in a dip in the tuning curve. **(B)** The oddball protocol. In the oddball protocol, two different stimuli are chosen (f1 and f2) so that both are within the response range of the neuron, giving rise to about the same response. One stimulus is chosen as the standard, being presented repeatedly for a long period. Within this sequence, the second stimulus, called the deviant, is embedded with a relatively low probability. Later, a second block is presented in which the roles are changed: the stimulus that was standard is now deviant, and vice versa. At the end of the experiments, the average responses to the standards are compared with the corresponding responses to the deviants. In non-specific adaptation, we expect no significance differences (left histogram). In SSA, we expect the responses to the deviant to appear consistently above the responses to standards (right histogram). **(C)** The constant order protocol. Two stimuli are presented in a long sequence, starting with several repetitions of f1 then switching to several repetitions of f2, and so on, alternating between the two stimuli. In non-specific adaptation, we expect that the response to the first stimulus in each sub-sequence will be adapted and therefore not larger than the response to the same stimulus when it is last in the sequence. In SSA, we expect a stronger response every time the sequence is switched from one stimulus to the other (right histogram in **C**). Modified from Gutfreund (2012).

Neural correlates of a particular behavior can be recorded at different levels, ranging from a single neuron to scalp-recorded EEG and fMRI. For example, in scalp-recorded potentials, an auditory component was found that shares some similarities with the phenomenon of habituation. This component is known as mismatch negativity (MMN; Naatanen, 1995). MMN is measured by presenting a sequence of auditory stimuli in which rare sounds or deviants are embedded occasionally. This type of stimulation paradigm is called oddball stimulation (Naatanen, 1995; Ulanovsky et al., 2004; see **Figure 4B**). In such conditions, the evoked potential to the deviant is usually stronger than the evoked potential to the standard. This phenomenon is believed to reflect a habituation-like process whereby the standard stimulus is memorized, allowing the detection and enhancement of responses to deviation from this memory (Nelken, 2004). However, scalp-recorded potentials reflect a global, indirect signal and is therefore limited in its ability to reveal fine details of the neural circuits that compute and represent habituation. For this purpose, identifying neural correlates of habituation at the single-neuron level is preferable.

Habituation is defined as a decline in behavioral response to a sustained or repeatable stimulus that is not fatigue (Thompson, 2009). At the single-neuron level, a reminiscent decline in the neural response to sustained or repeatable stimulus, called adaptation, is a ubiquitous property of sensory neurons (Westerman and Smith, 1984; Gutfreund and Knudsen, 2006). However, it is important to emphasize that habituation is not a mere reduction in behavioral response with time. As described above, habituation is a learning process in which a standard or rather unchanging background scene is learned in order to allow an animal to respond selectively to behaviorally relevant stimuli (**Figure 1**; Barry, 2009). Thus, not all types of neural adaptations can serve as neural correlates of habituation. Two major types of adaptation have been described in the literature. One is the non-specific adaptation that depends on the history of activation of the neuron more than on specific features of the stimulus (Calford and Semple, 1995; Brosch and Schreiner, 1997; McAlpine et al., 2000; Ingham and McAlpine, 2004; Furukawa et al., 2005; Wehr and Zador, 2005; Gutfreund and Knudsen, 2006). The other is stimulus-specific adaptation (SSA), an adaptation to a specific stimulus that does not generalize to other stimuli, regardless of how active the neuron was (Ulanovsky et al., 2003, 2004; Antunes et al., 2010). SSA is of particular interest here since, similar to habituation, it depends on the history of the environment rather than on the activity of the neuron. Indeed, the SSA phenomenon has been called single-neuron habituation (Nelken and Ulanovsky, 2007). To distinguish SSA from non-specific adaptation, a variety of stimulation paradigms have been used (**Figure 4**). In all of them a standard stimulus is presented several times in a row to induce adaptation. In SSA the responses to stimuli that are physically close to the standard are expected to be more reduced compared to stimuli that are different, whereas in non-specific adaptation the responses to all stimuli are expected to be reduced. In the priming protocol (**Figure 4A**), if SSA exists we expect a dip in the tuning curve near the standard stimulus, whereas if the adaptation generalizes (non-specific) we expect the whole tuning curve to attenuate. In the oddball paradigm (**Figure 4B**), the responses to standard (common) stimuli are compared with responses to the same stimuli when presented rarely (as deviants). SSA implies that the neuron’s response to the common stimulus and not to the deviant stimulus is reduced, and, as a result, the response to the deviant stimulus is stronger compared to the same stimulus when presented commonly. In the constant order paradigm (**Figure 4C**), we look at the points of shift between one stimulus type to the other. If SSA exists we expect that the responses to the first stimuli after the shift will be less affected by previous stimuli (because previous stimuli are different), and therefore will be larger compared to subsequent stimuli where previous stimuli are the same. The stimulation protocols illustrated in **Figure 4** may provide an experimental framework for seeking neural correlates of habituation.

Using such protocols, it was shown that SSA is a common phenomenon in the brain and has been observed in visual (Muller et al., 1999), somatosensory (Katz et al., 2006), and auditory (Perez-Gonzalez et al., 2005) pathways. SSA was studied in greater detail in the auditory system of cats, rats, and monkeys (Ulanovsky et al., 2003; Perez-Gonzalez et al., 2005; Fishman and Steinschneider, 2012). Neurons sensitive to deviations were found at different levels of the auditory pathway, namely, in the inferior colliculus (IC), the auditory thalamus, and the auditory cortex (Ulanovsky et al., 2004; Anderson et al., 2009; Malmierca et al., 2009; Antunes et al., 2010; Farley et al., 2010; Ayala and Malmierca, 2013; Ayala et al., 2013). SSA is measured in anesthetized as well as awake animals (Richardson et al., 2013) and is therefore pre-attentive. Detailed characterization of SSA in the auditory system revealed that it is highly sensitive to minute deviations from the standard frequency. In the auditory cortex of cats, neurons have been found to respond significantly stronger to stimuli that are deviant from the standard by a frequency difference as small as 0.1% (Ulanovsky et al., 2003). Moreover, it was shown that this adaptation has several time scales ranging from sub-seconds to 10s of seconds (Ulanovsky et al., 2004). This implies that the expected model of the background can be updated on a fast time scale to encompass rapid changes in the stimulus environment, but at the same time, a longer history of stimulation is allowed to affect subsequent responses.

In summary, recent studies on auditory SSA in the cortex, the thalamus and the IC suggest that at the single-neuron level, habituation-like responses are widespread. The origin and mechanisms of this phenomenon are yet to be discovered. And, not less important, the questions of if and how this phenomenon at the single-neuron level is related to habituation at the behavioral level must be answered. Three major problems discussed below hinder the attempts to relate SSA to habituation.

### THE MULTIPLE FEATURE PROBLEM

Stimulus-specific adaptation in the auditory cortex and the IC has been studied mostly using pure tone stimuli where the deviants differed from the standards in terms of sound frequency. Other sensory features such as stimulus intensity, stimulus location, or stimulus length were either not studied or gave rise to poor SSA (Ulanovsky et al., 2003; Farley et al., 2010). An exception is a very recent study in which it was shown that SSA exists in the auditory cortex of rats between stimuli of similar frequencies but differ in their temporal noise structure (Nelken et al., 2013). In nature, a stimulus can differ from what has been in the past along multiple features, i.e., frequency, amplitude, duration, location, etc., or combinations of features. A neuron that is sensitive to changes in the frequency of the stimulus but not to changes in other features is a limited “change detector” that cannot explain the general sensitivity to deviant stimuli observed behaviorally. It is therefore necessary to identify types of SSA that encompass multiple sensory features. Moreover, the phenomenon of habituation is amodal, independent of sensory modality (Thompson and Spencer, 1966). Neural correlates of habituation should therefore not be limited to auditory neurons.

### THE MEMORY TRACE PROBLEM

The memory trace of adaptation is the time it takes from the last stimulus until its effect on the response to the next stimulus wears out. In the laboratory, this is measured by presenting sequences of stimuli with various inter-stimulus time intervals (ISIs). The minimal ISI in which no adaptation occurs is the duration of the memory trace. SSA in the auditory cortex, the thalamus or the IC has been reported at ISIs as long as 2 s (Ulanovsky et al., 2003; Ayala and Malmierca, 2013) or has only been studied at ISIs < 2 s (von der Behrens et al., 2009; Antunes et al., 2010). Therefore, we can conclude that information about the standard is stored in memory for about 2 s. This poses a major problem because many examples of behavioral habituation have been reported with ISIs of 10s of seconds to minutes, even for short duration stimuli (Thompson and Spencer, 1966; Weinberger et al., 1975; Valentinuzzi and Ferrari, 1997; Bala and Takahashi, 2000; Zimmer, 2006; Dong and Clayton, 2009; Glanzman, 2009). It is therefore necessary to identify a form of SSA that maintains a longer memory trace.

### CHANGE DETECTION VERSUS PROBABILITY DETECTION

Most studies of SSA in the auditory pathways were conducted using standard oddball paradigms whereby a deviant frequency is embedded with a certain probability in the sequence of standard frequency (**Figure 4B**). In such a probabilistic stimulus, SSA implies that the response of the neuron is modulated by the probability of the deviant; the smaller the probability, the larger the response. But sensitivity to probability is not necessarily equal to habituation. Habituation requires learning an expectation rule set by the standard and pointing out any deviations from this rule, while sensitivity to probability simply requires counting the number of stimuli over a period of time and responding accordingly. Rare stimuli are not always salient. For example, a stimulus can appear in a sequence of multiple different stimuli, each being rarely presented, but none is salient compared to the others (Taaseh et al., 2011). The standard oddball paradigm cannot distinguish between the two possibilities. Despite recent attempts to resolve this issue for SSA in the auditory cortex, it still remains an open question (Farley et al., 2010; Taaseh et al., 2011).

In summary, we have provided a short review of the phenomenon of SSA in the auditory system and argued that SSA is the closest reported phenomenon at the single-neuron level to act as a neural correlate of habituation. However, by itself, the well-studied type of SSA in the main auditory pathway of mammals is not sufficient to account for habituation. We therefore now return to the OT/SC. If, as suggested above, the OT/SC is involved in saliency mapping and habituation, we expect to find a new type of SSA in this structure that is sensitive to multiple stimulus features and modalities and has a longer memory trace.

## NEURAL ADAPTATION IN THE OT/SC

### NEURAL ADAPTATION IN THE OT/SC IS STIMULUS-SPECIFIC

**FIGURE 5:**
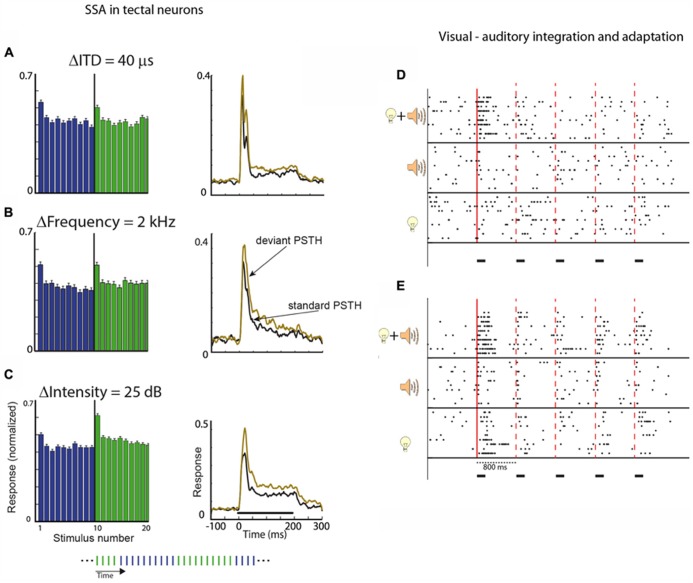
**(A)** The histogram shows the average response of a population of neurons in the OT to a long sequence of stimuli (200 stimuli). The duration of each stimulus was 200 ms and the ISI was 1 s. The sequence alternated between two ITD values; every 10 repetitions the ITD of the sound was switched to the other value (protocol is illustrated in the inset at the bottom of the figure). The graph on the right shows the post-stimulus time histogram (PSTH) of one stimulus when it was first in its sub-sequence (deviant, light red curve) compared to the PSTH for the same stimulus when it was last in its sub-sequence (standard, black curve). **(B)** Same as **A**, but in this case, the two stimuli were narrowband stimuli (width of 1 kHz) differing in their central frequency. **(C)** Same as **A** and **B**, but in this case, the two stimuli differed in intensity. One stimulus (dark blue bars) was softer compared to the other (light red bars). The average response to deviant stimulus was greater in all cases. Modified with permission from Reches and Gutfreund (2008). **(D,E)** Two examples of tectal recordings showing bimodal enhancement. The raster plots show results of unit responses to a sequence of five repetitions of a stimulus with an ISI of 800 ms. The time course of stimulation is designated by the horizontal black bars. Stimuli were either five visual stimuli, five auditory stimuli, or five congruent bimodal stimuli (visual and auditory stimuli appearing together from the same location). The solid vertical line designates the onset of the first stimulus in the sequence. The dashed vertical lines designate the onset of subsequent stimuli. In both examples **(D,E)**, stronger responses to bimodal stimuli can be seen in the response to the first stimulus in the sequence compared to the unimodal responses. This bimodal enhancement is missing in the responses to the subsequent stimuli. Modified from Zahar et al. (2009).

Neural adaptation to visual stimuli is robust in the OT, particularly in the deep layers (Woods and Frost, 1977; Boehnke et al., 2011). Habituation-like responses, i.e., SSA, were reported in the OT of pigeons. Some neurons were shown to lose responses completely to repeated visual stimuli, but changes in the type of stimuli resulted in a return of the response to the initial level (Woods and Frost, 1977). Adaptation to visual stimuli in the SC was also reported in the monkey (Fecteau and Munoz, 2005). Recently, adaptation to auditory stimuli was characterized systematically in the barn owl’s OT (Reches and Gutfreund, 2009; Netser et al., 2011). Oddball and constant order protocols (see **Figures 4B,C**, respectively) were used. It was found that most neurons in the OT responded more strongly to a low probability stimulus than to a high probability stimulus (in the oddball protocol) or more strongly to the first stimulus, which is different from its past compared to the last stimulus (in the constant order protocol). For example, neurons in the OT of the owl are tuned to the interaural time difference (ITD) of the sound, which is the major localization cue for the horizontal position. By presenting sounds through ear phones, it is relatively easy to manipulate the ITD of the sound delivered to the animal (Moiseff and Konishi, 1981). **Figure 5A** shows the average results from constant order experiments in which the stimulus sequence alternated every 10 stimuli between two ITD values (see **Figure 4C**). It can be seen that every time a switch occurred between one ITD to the other, the response increased and adapted again until the next switch occurred. By comparing the response to a stimulus when it is first in its sequence with the response to the same stimulus when it is last in its sequence, it is possible to quantify the SSA. Remarkably, significant changes in neural responses between the first and last stimuli were observed even when the two stimuli differed by an ITD difference as small as 20 μs (Reches and Gutfreund, 2008). Thus, the neural response to an auditory stimulus depends not only on the value of the ITD of the stimulus but also on how this value differs from previous ITD values.

Sensitivity to changes in the input stream was not limited to changes in the ITD of the sound. Testing for changes in the frequency, intensity, or interaural level difference (ILD) of the sound all gave rise to the same basic result. The neurons readily responded more strongly to changes in the input stream along all stimulus dimensions (Reches and Gutfreund, 2008). In all cases (ITD, ILD, intensity, and frequency), SSA was evident and developed rapidly after one trial (**Figures 5A–C**). This similarity between the different features is especially striking, taking into account that the four features are represented and computed in markedly different ways but all still exhibit the same SSA. ITD and ILD, the two primary binaural localization cues, are processed in parallel in two separate and independent brainstem pathways (Takahashi et al., 1984; Takahashi and Konishi, 1988; Adolphs, 1993; Albeck and Konishi, 1995); frequency separation is maintained in the ascending auditory pathways from the cochlea up to the level of the lateral shell of the IC where information is combined across frequency-specific channels (Euston and Takahashi, 2002). Sound-level information is presumably manifested in the firing rates of neurons in the ascending pathways. Therefore, the fact that all four independent acoustic features showed a qualitatively similar pattern of adaptation suggests that SSA is an important property in the neural representation of the auditory scene in the OT. This property possibly underlies the owl’s ability to attend and orient abruptly to novel events. For comparison, similar tests were performed in the IC, the main source of auditory information to the OT. Significant SSA in the IC was found only to the frequency of the sound but not to other stimulus features such as ITD, ILD, and intensity (Reches and Gutfreund, 2008). Thus, robust multi-feature deviant detection develops in the OT and not earlier in the pathway.

The robustness of SSA in tectal neurons gives rise to an interesting ambiguity problem, i.e., the inability to discriminate between two conditions. For example, a soft sound usually produces weaker responses in the OT compared to a louder sound, however, the same soft sound can induce stronger neural responses than the loud sound when it is deviant in an environment characterized by loud sounds (Reches and Gutfreund, 2008). Thus, neural responses in the tectum seem unable to code the sensory identity of the incoming sound unambiguously. This, together with the finding that OT/SC neurons are mostly broadly tuned to sensory features such as frequency, amplitude modulations, orientation, direction, and modality (Mize and Murphy, 1976; Zahar et al., 2009) is consistent with the hypothesis that the OT represents the location of the stimulus and how salient it is. The exact identification of the stimulus is not a computational task of OT, presumably carried out in a different pathway.

### MULTISENSORY INTEGRATION IN THE OT/SC ENHANCES DEVIANCE DETECTION

In the laboratory, saliency mapping is usually studied in unimodal settings, however, in nature it is primarily an amodal task. The saliency of an event is determined by a combination of modalities (Stein and Meredith, 1993; Pluta et al., 2011), therefore we expect multisensory integration to take place in the saliency mapping pathways. Indeed, multisensory integration is a hall-mark of the OT/SC (Stein and Meredith, 1993). Visual, auditory, and somatosensory inputs converge onto the SC, resulting in multisensory neurons that integrate information between modalities (Meredith and Stein, 1986; Meredith et al., 1987). When a visual and auditory stimuli are presented from the same location and at the same time (congruent bimodal stimulation), many neurons enhance their responses dramatically compared with the responses to the visual or auditory stimulus alone. This phenomenon, known as multisensory enhancement, has been characterized in great detail in the SC of cats and monkeys (Wallace et al., 1996; Stanford et al., 2005). Recently, multisensory enhancement was studied in barn owls using paradigms that allow testing for adaptation as well. In a simple adaptation paradigm where the same stimulus was repeated several times (Zahar et al., 2009), it was found that multisensory enhancement of the first stimulus presented before adaptation was robust and comparable to what has been reported in mammals. However, subsequent stimuli presented after adaptation did not show clear multisensory enhancement (**Figures 5D,E**; Zahar et al., 2009). A similar result was shown using oddball stimuli; the multisensory enhancement was much stronger when the stimuli were deviant in the sequence compared to when the stimuli were common (Reches et al., 2010). Thus, multisensory enhancement in the OT is able to increase deviance detection. The mechanisms of this phenomenon are not clear. However, it supports the idea that multisensory integration is used by the OT to enhance saliency mapping (Pluta et al., 2011) by enhancing SSA in congruent bimodal settings (Reches et al., 2010).

### THE MEMORY TRACE OF ADAPTATION IN THE OT IS RELATIVELY LONG

A common notion in the adaptation of neural responses, backed up by computational models of synaptic suppression (Tsodyks and Markram, 1997), is that the dynamics of neural adaptation complies with stimulus duration (Marom, 2009): a short duration stimulus is expected to induce short-lasting adaptation, and vice versa (Varela et al., 1997; Ulanovsky et al., 2004). This concept is in line with most studies of auditory SSA described above. In these studies, the ISIs varied between 300 ms and 2 s, stimulus durations were in the range of 100–500 ms, and the probability of the deviant was 10–15%. None of the papers cited above reported a memory trace longer than 2 s, which is within the time scale of the stimulus timing. However, as mentioned earlier, this relatively short memory trace constitutes a major problem for linking neural SSA with mechanisms of habituation. Behavioral habituation does not comply with the above-mentioned principle of comparable time scales. For example, in the barn owl, reflexive pupil dilation and eye movements to sequences of relatively short stimuli with ISIs of 10–13 s readily habituated and recovered when the stimulus was switched from one type to the another (Bala and Takahashi, 2000; Spitzer et al., 2003; Netser et al., 2011).

To close the time gap between behavioral habituation and SSA in the OT, it is important to examine the memory trace of the SSA. This was done in the OT of barn owls by presenting sequences of identical sounds and measuring unit responses as a function of the position of the stimulus in the sequence. Stimuli with ISIs of 10, 30, and 60 s were tested. Remarkably, at all ISIs tested, a single, short (300 ms), and weak (20 dB above the unit’s threshold) stimulus was sufficient to induce a significant reduction in the neural response to the second stimulus in the sequence (Netser et al., 2011). Moreover, just like in habituation, the system not only memorized that there was a stimulus earlier but also what type of stimulus. For example, when presenting three short auditory stimuli with an ISI of 60 s, the first two being identical and the third different, the response to the second stimulus was reduced compared to the first, but the response to the odd third stimulus, 60 s later, was completely recovered (Netser et al., 2011). The finding that the memory trace of specific adaptation in the OT can reach time spans of over a minute suggests that this type of adaptation is a neural correlate of behavioral habituation, further supporting the link between tectal neural circuitry and habituation.

## FINAL REMARKS

Habituation is commonly known as a reduction in behavioral responses to repeated stimuli. However, the scope of habituation is broader. A seminal work by Sokolov (1963) conceptualizes habituation as a process of learning and memorizing the background for the selection of incoming stimuli that are odd from the background. In this review, we aimed at emphasizing this sometimes forgotten aspect of habituation: the ability to select incoming stimuli if they do not match the stored representation of the background. We reviewed here evidence that the SC in mammals and the OT in birds are involved in the stimulus selection process. In addition, we reviewed recent results suggesting that the activity of tectal neurons may be correlated with habituation of the orienting response. Thus, combined together, these results point to the tectal/colliculi circuitry as a promising model system for studying habituation mechanisms.

In recent years, the avian OT has emerged as a model for studying the neural mechanisms of saliency mapping in space (stimulus competition) and time (habituation; Marin et al., 2005, 2007, 2012; Wang et al., 2006; Reches and Gutfreund, 2008; Lai et al., 2011; Mysore and Knudsen, 2011b, 2013; Netser et al., 2011). The advantage is that the midbrain circuitry in birds is highly segregated and experimentally accessible (Knudsen, 2011). The overall findings are strikingly similar to findings in other species, including primates. This similarity stresses the importance of a comparative approach to gain an evolutionary perspective on basic elements of animal behavior such as habituation and attention. Here, we focused on recent studies in barn owls regarding SSA in the OT and its possible link to habituation. Yet, two important questions remain to be answered:

### WHAT ARE THE NEURAL MECHANISMS OF SSA IN THE OT?

The neural mechanisms underlying SSA in the auditory pathways as well as in the OT are unknown. One common model to explain SSA is that different stimuli activate separate paths to the recorded neuron and that basic adaptation mechanisms (synaptic depression or intrinsic cellular mechanisms) act at levels where the activation is separated (Eytan et al., 2003). An intriguing observation in the barn owl’s SSA is a complete lack of cross-stimulus adaptation at long ISIs, even though the frequency content of the two stimuli overlapped substantially (Netser et al., 2011). It is therefore unlikely that this type of SSA is accounted for solely by basic response suppressions at lower, frequency-specific levels. To compute the deviancy of complex broadband sounds, a network is required that compares the neural responses to the current stimulus with previous responses based on an integration of information about frequency and amplitude modulations. Details of such a network are yet to be discovered.

### WHAT IS THE RELATIONSHIP BETWEEN SSA AND BEHAVIOR?

The phenomenon of SSA has been studied mostly in anesthetized animals. It is yet to be shown what effects it has on behavior. As previously mentioned, not all types of SSA are linked necessarily to behavioral habituation and saliency mapping. Some may be related to scene analysis or optimal coding (reviewed in Winkler et al., 2009). One approach for studying the relationships between neural adaptation and behavior would be to record behavioral and neuronal responses simultaneously and examine the trial-by-trial correlations between SSA and behavioral habituation. Another approach would be to inactivate brain areas that contribute to SSA and examine the effects on behavioral habituation and on the animal’s ability to respond to changes in the environment. Future experiments on these directions are likely to shed light on the neural basis of habituation.

## Conflict of Interest Statement

The authors declare that the research was conducted in the absence of any commercial or financial relationships that could be construed as a potential conflict of interest.
